# Characterization of drug-induced human mitochondrial ADP/ATP carrier inhibition

**DOI:** 10.7150/thno.54936

**Published:** 2021-03-05

**Authors:** Stephany Jaiquel Baron, Martin S. King, Edmund R.S. Kunji, Tom J.J. Schirris

**Affiliations:** 1 Medical Research Council Mitochondrial Biology Unit, University of Cambridge, Cambridge Biomedical Campus, Keith Peters Building, Hills Road, Cambridge, CB2 0XY, United Kingdom.; 2 Department of Pharmacology and Toxicology, Radboud Institute for Molecular Life Sciences, Radboud Center for Mitochondrial Medicine, Radboud University Medical Center, Nijmegen, The Netherlands.

**Keywords:** drug-induced mitochondrial dysfunction, mitochondrial transport proteins, adenine nucleotide translocase, thermostability shift, transport inhibition kinetics.

## Abstract

An increasing number of commonly prescribed drugs are known to interfere with mitochondrial function, causing cellular toxicity, but the underlying mechanisms are largely unknown. Although often not considered, mitochondrial transport proteins form a significant class of potential mitochondrial off-targets. So far, most drug interactions have been reported for the mitochondrial ADP/ATP carrier (AAC), which exchanges cytosolic ADP for mitochondrial ATP. Here, we show inhibition of cellular respiratory capacity by only a subset of the 18 published AAC inhibitors, which questions whether all compound do indeed inhibit such a central metabolic process. This could be explained by the lack of a simple, direct model system to evaluate and compare drug-induced AAC inhibition.

**Methods:** For its development, we have expressed and purified human AAC1 (hAAC1) and applied two approaches. In the first, thermostability shift assays were carried out to investigate the binding of these compounds to human AAC1. In the second, the effect of these compounds on transport was assessed in proteoliposomes with reconstituted human AAC1, enabling characterization of their inhibition kinetics.

**Results:** Of the proposed inhibitors, chebulinic acid, CD-437 and suramin are the most potent with IC_50_-values in the low micromolar range, whereas another six are effective at a concentration of 100 μM. Remarkably, half of all previously published AAC inhibitors do not show significant inhibition in our assays, indicating that they are false positives. Finally, we show that inhibitor strength correlates with a negatively charged surface area of the inhibitor, matching the positively charged surface of the substrate binding site.

**Conclusion:** Consequently, we have provided a straightforward model system to investigate AAC inhibition and have gained new insights into the chemical compound features important for inhibition. Better evaluation methods of drug-induced inhibition of mitochondrial transport proteins will contribute to the development of drugs with an enhanced safety profile.

## Introduction

Mitochondria produce the majority of cellular ATP, and are a central hub for many metabolic pathways 1. The advent of molecular medicine has implicated mitochondria as major regulators of cell death 2 and in the initiation and propagation of innate immune responses and inflammatory reactions 3, 4. Consequently, their dysfunction is associated with many inherited and common disorders, including neurodegenerative, developmental and metabolic diseases, several cancers, type II diabetes, and cardiovascular disorders 1, 5-9.

Moreover, a steadily increasing number of commonly prescribed drugs are known to interfere with mitochondrial function, including cholesterol-lowering and anti-diabetic drugs, antibiotics, chemotherapeutics and immunosuppressants 10-14. The relevance of drug-induced mitochondrial dysfunction in adverse drug effects is exemplified by the observation that it associates with approximately 50% of all FDA black box warnings 15. In addition, a screen of 676 unique compounds demonstrated that 73% negatively affected mitochondrial function 16.

Although drugs can potentially interfere with the function of all ~1200 mitochondrial proteins 17, mitochondrial drug off-target mechanisms are generally categorized as inhibition of oxidative phosphorylation, respiratory uncoupling, permeability transition pore opening, suppression of fatty acid β-oxidation, and affected mitochondrial DNA replication, transcription or translation 18. Although often not considered, mitochondrial transport proteins form another significant class of potential mitochondrial off-targets, as they account for more than five percent of the mitochondrial proteome. Moreover, they have a pivotal role in mitochondrial metabolism, as they facilitate the transport of metabolites, inorganic ions and cofactors across the largely impermeable mitochondrial inner membrane, linking the metabolic pathways of the mitochondrial matrix with those in the cytosol 1, 19, 20. This central role in cellular metabolism is emphasized by the association of various inborn mitochondrial diseases with mitochondrial transport protein deficiencies 19.

Most of these transporters belong to the mitochondrial carrier family, consisting of 53 members that enable import and efflux of a wide variety of metabolites 1, 19, 20. Other types of transport proteins that are embedded in the mitochondrial inner membrane include ATP-binding cassette (ABC) transporters 21, various ion-channels, sideroflexins 22, 23 and the mitochondrial pyruvate carrier 24, 25. Expression patterns vary greatly between different carriers, ranging from ubiquitous to organ-specific expression 26-28. Although mitochondrial carriers are involved in the import and export of a large variety of chemically distinct cellular metabolites, they are likely to have a similar mechanism of transport, which has recently been elucidated for the mitochondrial ADP/ATP carrier (AAC) 1, 29.

The AAC is the canonical member of the SLC25 mitochondrial carrier family, importing ADP from the cytosol in exchange for mitochondrial ATP 29-32. Consequently, this carrier mediates an essential step in eukaryotic oxidative phosphorylation 29-32. The carrier is composed of three homologous domains, each of which contains two transmembrane alpha-helices linked by a hydrophilic loop with a small helix facing the matrix space; both the N- and C-terminal regions extend into the intermembrane space of mitochondria 33. The transport mechanism requires the carrier to cycle between cytoplasmic- and matrix-open states, alternating the accessibility of the central substrate binding site to these compartments 29, 34. There are four human AAC isoforms expressed from different genes (SLC25A4, SLC25A5, SLC25A6 and SLC25A31) 35. The carriers cycle between a cytoplasmic-open and matrix-open state via an occluded state, which is closed on both the matrix and cytosolic side 29-31, 34. The differences in tissue-specific transcription of these isoforms are thought to be associated with the distinct energy requirements of various cell types in different states of cellular differentiation 35-37.

The central role of this carrier in energy metabolism makes it a potentially important off-target underlying drug-induced mitochondrial dysfunction. The first report linking AAC inhibition to mitochondrial dysfunction dates to the 1960s, describing the toxic effects of atractyligenin, a coffee bean extract and precursor of the most potent canonical AAC inhibitor carboxyatractyloside (CATR) 38. Mitochondrial ADP-uptake inhibition by the diuretics ethacrynic acid and furosemide 39, illustrate the first cases of AAC inhibition by commonly used drugs. AAC inhibition has also been observed with various other commonly used drugs, including several non-steroid anti-inflammatory drugs (aspirin, diclofenac, indomethacin, and nimesulide), the anti-depressant sertraline, drugs used to treat psoriasis (anthralin and tretinoin), the antibiotic equisetin, and the antiretroviral drug zidovudine 40-47. Toxicity of two potential anti-obesity drugs (ibipinabant and leelamine) was associated with AAC inhibition in preclinical drug development studies 48, 49. More recently, AAC inhibition has been explored as an anti-cancer therapy, as isoform 2 has been associated with cancer cell metabolism 36. This observation has initiated the exploration of novel AAC inhibitors, including CD-437, and two flavonoids (quercetin and apigenin) 49-54. In this context, specific AAC2 inhibition was shown for chebulinic acid and suramin, the latter of which is a treatment for African sleeping sickness 55. Moreover, inhibition of isoform 4 has recently been explored as a novel therapeutic strategy for male contraception, as this isoform is expressed in male reproductive tissues 56.

Previously used methods to detect drug-induced AAC inhibition often involve complex whole cell- or mitochondria-based assays, or overexpression of human isoforms in the Gram-positive bacterium *Lactococcus lactis* 49 and in the yeast *Saccharomyces cerevisiae* 49, 55*.* Alternatively, human AAC2 has been expressed in inclusion bodies, refolded and reconstituted into proteoliposomes, which were used to study the inhibitory effects of chebulinic acid and suramin 55. The use of a wide range of methodologies, as well as indirect model systems, limits the direct comparison of the proposed inhibitors, but more importantly, any inhibitory effects may be due to indirect effects, mediated via other mitochondrial off-target mechanisms. The use of purified, detergent-solubilized human AAC could overcome this limitation, but the yield is low when isolated from human mitochondria 57. Similar problems with protein yield have hampered the expression of human AAC isoforms in bacteria and yeast cells 58-60.

Here, we describe the expression in *Saccharomyces cerevisiae*, purification and functional reconstitution of functional human AAC1 (hAAC1), the main isoform present in heart and skeletal muscle 35, 37. We use thermostability shift measurements of hAAC1 to investigate the binding of these compounds, demonstrating its advantages and limitations as a high-throughput screening method to identify potential adverse drug interactions with the carrier. Finally, the effect of these compounds on transport was assessed in proteoliposomes, enabling direct characterization of the inhibition kinetics.

## Materials and Methods

### Chemicals

Equisetin, CD-435 and ibipinabant ((*S*)-SLV 319) were purchased from Cayman Chemical Company. Leelamine was purchased from Toronto Research Chemicals. All other chemicals were purchased from Merck, unless otherwise stated.

### Cell culture

HeLa cells (American Type Culture Collection, CCL-2) were maintained at 37 °C in a humidified atmosphere of 5% (*v/v*) CO_2_ in Dulbecco's Modified Eagle's medium (DMEM) containing 25 mM D-glucose, GlutaMAX^TM^, 1 mM pyruvate (Gibco^TM^ Life Technologies) supplemented with 10% (*v/v*) Fetal Bovine Serum (FBS, Gibco^TM^ Life Technologies) and split to maintain cellular confluency below 90% in 75 cm^2^ cell culture vessels (Corning).

### Cellular and mitochondrial oxygen consumption analysis

Analysis of cellular and mitochondrial respiratory capacity was performed using the Seahorse XF96 extracellular flux analyzer (Agilent). HeLa cells were counted using a Countess automated cell counter (ThermoFisher Scientific), and seeded in 96-well cell culture plates 24-h prior to the experiment at a 14,000 cells per well density. We used cells with passage numbers ranging between 14 and 18, to minimize the contribution passage-dependent metabolic changes. Culture medium was replaced one hour before initiation of the experiment by sodium bicarbonate free DMEM medium, pH 7.4, supplemented with 1 mM pyruvate, 25 mM D-glucose, 32 mM NaCl and 10 mM HEPES. Cells were allowed to reach a stable routine oxygen consumption, after which the compounds were added (final DMSO concentration 0.1% (*v/v*)). Subsequently, oxygen consumption was measured for 90 min, after which we continued using 'cell mito stress test' protocol as described previously 61. To determine mitochondrial oxygen consumption rates, culture medium was removed and washed once with mitochondrial assay solution (MAS; pH 7.2, 70 mM sucrose, 220 mM mannitol, 10 mM KH_2_PO_4_, 5 mM MgCl_2_, 2 mM HEPES, 1 mM EGTA, 0.2% (*w/v*) essentially fatty acid-free bovine serum albumin). Subsequently, cells were permeabilized 20 min prior to initiation of the experiment by the addition of MAS containing 10 μg • mL^-1^ digitonin, 10 mM glutamate, and 10 mM malate. Cells were allowed to reach stable non-stimulated respiration, after which the compounds were added (final DMSO concentration 0.1% (*v/v*)). Subsequently, oxygen consumption was measured for 90 min, after which 4 mM ADP was added to determine maximal ADP-stimulated respiration. 1 μM rotenone (final DMSO concentration 0.04% (*v/v*)) and 2.5 μM antimycin A (final DMSO concentration 0.04% (*v/v*)) were added simultaneously to obtain non-mitochondrial respiration, as described previously 62. For both cellular and mitochondrial oxygen consumption, non-mitochondrial respiratory rates were subtracted from all respiratory rates. Next, all rates were normalized to the average of the first three data points (T_0_).

### Expression of human AAC in *Saccharomyces cerevisiae*

Human ADP/ATP carrier isoform 1, codon-optimized for expression in *Saccharomyces cerevisiae* (GenScript), was truncated using PCR, resulting in a construct encoding residues 11-298 (hAAC1Δ1-10). The AAC2 from *Saccharomyces cerevisiae,* encoding the full sequence was a generous gift from Dr. Jonathan Ruprecht 63. The yeast AAC2, complete hAAC1, and hAAC1Δ1-10 constructs were engineered to contain an N-terminal tag composed of eight histidine residues and a Factor Xa protease cleavage site, and cloned into a pYES3/CT vector (Invitrogen) with a constitutively active promoter (pMIR) as described previously 34. Sequence-verified plasmids were transformed into *Saccharomyces cerevisiae* strains WB.12 (MATa *ade2-1 trp1-1 ura3-1 can1-100 aac1::LEU2 aac2::HIS3*) 64 and W303-1B (MATα leu2-3,112 trp1-1 can1-100 ura3-1 ade2-1 his3-11,15) using the LIAc/SS carrier DNA/PEG method 65. Transformants were selected on Sc-Trp + 2% (*w/v*) glucose plates. For large scale expression, a pre-culture of cells grown in Sc-Trp + 2% (*w/v*) glucose was inoculated into 100 L of YEPG medium in an Applikon Pilot Plant 140 L bioreactor. Cells were grown at 30 °C for 24 h and harvested by centrifugation (4,000 *g*, 20 min, 4 °C). Crude mitochondria were prepared using a bead mill (Dyno-Mill Multilab, Willy A. Bachofen AG) by established methods 66.

### Functional complementation of *Saccharomyces cerevisiae* AAC knockout strain

The rescue of non-fermentative growth of WB.12 by expression of ScAAC2, hAAC1, and hAAC1∆1-10 was assessed on YPG media. Transformant cells from glycerol stocks were inoculated into 5 mL Sc-Trp + 2% (*w/v*) glucose and incubated overnight at 30 °C with aeration at 225 rpm. The cells were pelleted by centrifugation (3,000 *g*, 10 min, 4 °C), washed four times with sterile de-ionized water, resuspended in 1 mL of water and the OD_600_ was adjusted to one. Serial dilutions were carried out (10^5^ - 10^1^) cells were spotted onto YPG plates, and incubated at 30 °C for 6 days.

### Preparation of lipid for protein purification

Tetraoleoyl cardiolipin (18:1) powder was purchased from Avanti Polar Lipids (#840012C) and solubilized in 10% (*w/v*) dodecyl maltose neopentyl glycol (Anatrace) by vortexing for 4 h at room temperature to give 10 mg • mL^-1^ lipid in a 10% (*w/v*) detergent stock. The stocks were stored in liquid nitrogen.

### Purification of human AAC via nickel affinity chromatography

Approximately one gram of isolated crude mitochondria was solubilized in 1% (*w/v*) dodecyl maltose neopentyl glycol, protease inhibitors (Complete Mini EDTA-free protease inhibitor tablets, 1 tablet • 50 mL^-1^, Roche), 20 mM imidazole and 150 mM NaCl for 1 h by rotation at 4 °C. The insoluble material was separated from the soluble fraction by centrifugation (200,000 *g*, 45 min, 4 °C). Nickel sepharose slurry (1 mL, corresponding to 0.7 mL resin; GE healthcare) was added to the soluble fraction; the mixture was stirred at 4 °C for 1 h. The nickel resin was harvested by centrifugation (100 *g*, 10 min, 4 °C), transferred to a proteus 1-step batch midi spin column (Generon), and washed with 30 column volumes (CV) of buffer A (20 mM HEPES pH 7.0, 150 mM NaCl, 40 mM imidazole, 0.2 mg • mL^-1^ tetraoleoyl cardiolipin / 0.2% (*w/v*) dodecyl maltose neopentyl glycol) (100 *g*, 10 min, 4 °C); followed by 10 CV of buffer B (20 mM HEPES pH 7.0, 50 mM NaCl, 0.2 mg • mL^-1^ tetraoleoyl cardiolipin / 0.2% (*w/v*) dodecyl maltose neopentyl glycol) (100 *g*, 7 min, 4 °C). The nickel resin was resuspended in buffer and incubated with 20 mM imidazole, 30 µg factor Xa protease and 5 mM calcium chloride on a rotator at 4 °C overnight. The protein was eluted by centrifugation (500 *g*, 2 min, 4 °C); the concentration of the purified protein was then measured by spectrometry (NanoDrop Technologies) at 280 nm (extinction coefficient: 47,120 M^-1^ cm^-1^; protein mass: 33,019 Da). Imidazole and NaCl were removed using a midi PD-10 desalting column according to the manufacturer's instructions (GE Healthcare) at 4 °C.

### Thermostability analysis of the hAAC1 carrier and drug interactions

To determine protein stability and to screen drugs to identify potential hAAC1 inhibitors, the thiol-specific fluorescent probe *N*-[4-(7-diethylamino-4-methyl-3-coumarinyl)phenyl] maleimide (CPM) 67 and the Rotor-Gene-Q (Qiagen) were used, using a modified protocol 68. CPM stocks (5 mg • mL^-1^ in DMSO) were diluted to 0.1 mg • mL^-1^ and equilibrated with purification buffer B for 10 min in the dark, before mixing with 3 µg of purified protein and compound. The mixture was equilibrated for a further 10 min in the dark at 4 °C. As binding of inhibitors can be state-dependent (for example, CATR binds exclusively to the cytoplasmic-open state, and bongkrekic acid (BKA) binds exclusively to the matrix-open state), it was necessary to also add the substrate ADP (5 μM) to allow cycling between states. The fluorescent intensity was measured at 460 nm (excitation) and 510 nm (emission) from 25 ºC to 90 ºC with a ramp of 1 ºC every 15 s. Data analysis and determination of the apparent melting temperature of the protein (Tm) were carried out with the software provided with the instrument.

### Reconstitution of protein into liposomes

A mix containing *E. coli* polar lipid extract (#100600P, Avanti Polar Lipids), egg L-α-phosphatidylcholine (#890704 (EPC-609), Avanti Polar Lipids) and tetraoleoyl cardiolipin (#840012C, Avanti Polar Lipids) in a 15:5:1 (*w/w*) ratio was dried under a stream of nitrogen. The lipid mixture was washed with methanol, and dried as before. Lipids were re-hydrated in 20 mM HEPES pH 7.0, 50 mM NaCl and, when required, ATP was added to a final concentration of 1 mM. The detergent pentaethylene glycol monodecyl ether (C_10_E_5_) was added to a final concentration of 1.6% (*v/v*) and the lipids were solubilized by vortexing and incubated on ice for one hour until the lipid emulsion became clear, subsequently, 30 μg protein was added per sample. The pentaethylene glycol monodecyl was removed by multiple additions of SM-2 bio-beads (Bio-Rad). Five additions of bio-beads were made to the master mix every 20 min with inversion at 4 °C: four of 60 mg, and the final of 480 mg. The samples were incubated overnight at 4 °C with inversion. Bio-beads were removed by passage of the sample through empty micro-bio spin columns (Bio-Rad). For exposed conditions, drugs were internalized at various concentrations (0.1, 0.3, 1, 3, 10, 30 and 100 μM; concentration used indicated in each figure) by freeze-thaw (three cycles), and sealed proteoliposomes formed by 21 passages through a 0.4-μm filter (Millipore). The external substrate was removed and exchanged into buffer (20 mM HEPES, pH 7.0 and 50 mM NaCl) using a PD10 desalting column (GE Healthcare). When required, drugs were also added externally to the indicated concentration as described below.

### Transport assays of wild-type and mutant AACs

Transport assays were performed using the Hamilton MicroLab Star robot (Hamilton Robotics Ltd). 100 μL of proteoliposomes were loaded into the wells of a MultiScreen_HTS_ + HA 96-well filter plate (pore size 0.45-μm, mixed cellulose ester, Millipore (#MSHAN4B)). Uptake of radiolabeled [^33^P]-ATP (Hartmann Analytic) in the presence or absence of 10 μM or 100 μM compound was initiated by the addition of 100 μL buffer containing 1 μM [^33^P]-ATP. Uptake was stopped after 0, 10, 20, 30, 45, 60, 150 s and 5, 7.5 and 10 min by filtration and washing with 3 times 200 μL ice-cold buffer (20 mM HEPES, pH 7.0 and 50 mM NaCl). Levels of radioactivity was measured by adding 200 μL MicroScint-20 (Perkin Elmer) and measured using a TopCount scintillation counter (Perkin Elmer). Uptake curves were fitted according to the one-phase association model or Michaelis-Menten and initial rates were determined from the linear part of the uptake curves. Transport measurements were also carried out in presence of different concentrations of the investigated drugs (0.1, 0.3, 1, 3, 10, 30 and 100 μM), added both internally and externally. The percentage of transport activity was plotted against the corresponding log_10_ inhibitor concentration in order to determine IC_50_-values of the tested compounds, the inhibitor concentration that caused a 50% reduction of the maximum transport activity.

### Molecular surface and charge calculations

2D structures of all compounds were drawn in MarvinSketch 20.15.0 (ChemAxon), and protonation was adjusted to pH 7.0. Next, 3D structures were generated using Avogadro 1.2.0 69 and energy minimization was performed according to the MMFF94 protocol with a steepest descent algorithm and 10 steps per update. Van der Waals surfaces (0.18 Å resolution), with electron density visualizations, were calculated using Avogadro's surfaces plugin. Solvent accessible surfaces (hydrophobic, polar, positively charged and negatively charged) and partial charges were calculated using MarvinSketch. BKA and CATR structures were obtained from their respective crystal structures PDB: 6GCI and PDB: 4C9G, published previously 29, 63.

### Quantification and statistical analyses

For CPM-based thermostability analysis, the apparent melting temperature (Tm) was calculated from the first derivative of the thermal denaturation profile. The average apparent Tm of 'no addition' control samples was subtracted from the apparent Tm measured for each compound addition in the same run, which provides the ΔTm. Statistical significance between groups was assessed by one-way ANOVA followed by Dunnett's *post hoc* analysis to correct for multiple comparisons test were performed using in Prism 8 (GraphPad Software, USA). Transport uptake curves (**Figures 2F**) were fitted with a one-phase association curve and dose-response curves (**Figure 4F-E**) using a sigmoidal dose-response regression curve-fitting. All values are shown as mean ± SEM, unless indicated otherwise.

## Results

### Metabolic profiling of proposed AAC inhibitors indicates mitochondrial dysfunction and reduced ADP-dependent respiration

**Figure 1:**
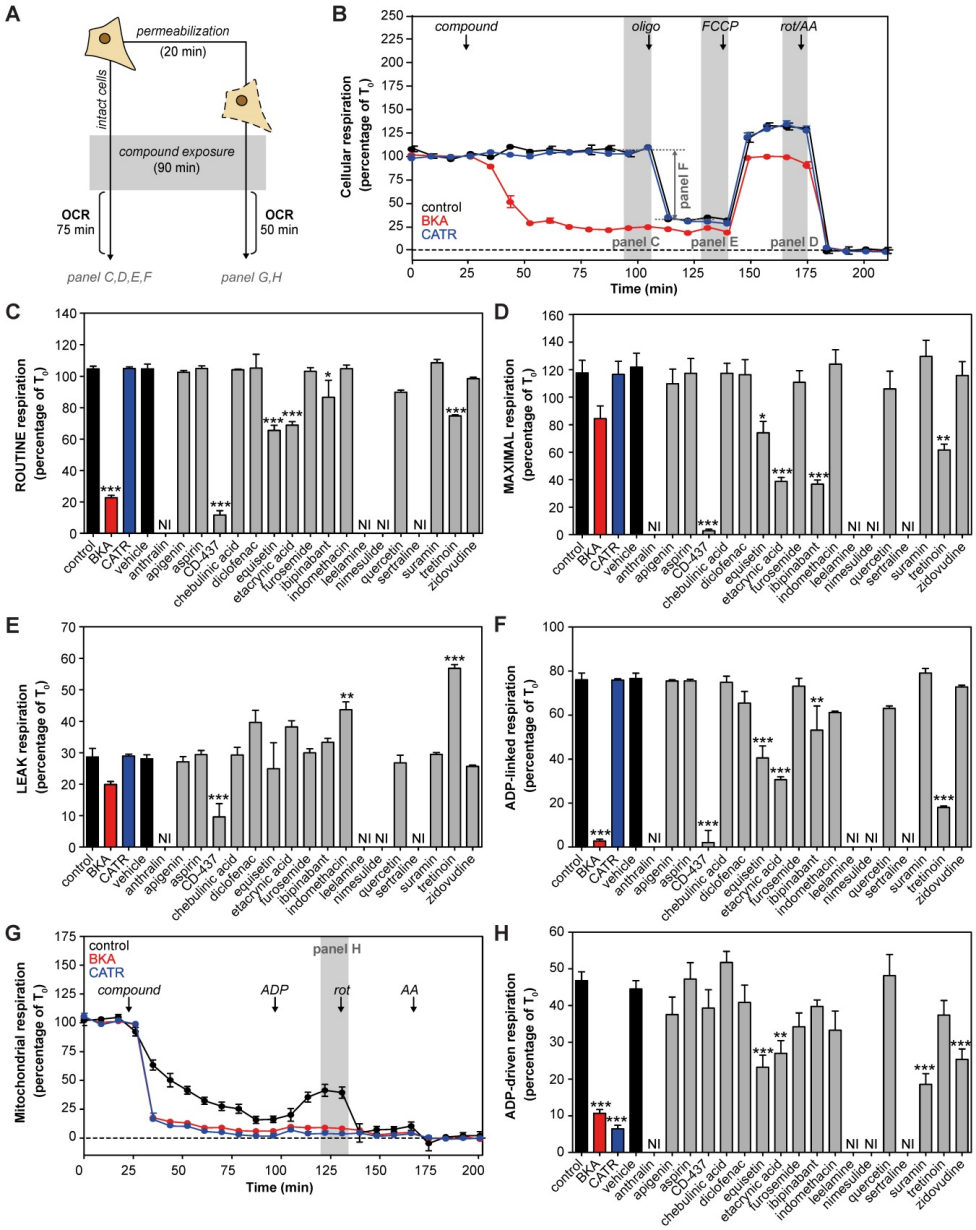
**| Cellular metabolic profiling of proposed AAC inhibitors. (A)** Effects of eighteen proposed AAC inhibitors on oxygen consumption rates (OCR) were investigated at 100 μM after 90 min exposure in intact and permeabilized cells. **(B)** For determination of cellular oxygen consumption rates, routine oxygen consumption was allowed to reach a stable signal (T_0_), which was used for normalization of all oxygen traces. Before normalization, non-mitochondrial respiration was determined after addition of rotenone (rot) and antimycin A (AA), and subtracted from all traces. Canonical AAC inhibitors bongkrekic acid (BKA, red circles and bars) and carboxyatractyloside (CATR, blue circles and bars) were used as positive controls. **(C)** Compounds were added and routine oxygen consumption was determined. NI: not interpretable, due to interference of the compounds with the fluorescent measurements. **(D-E)** The mitochondrial complex V inhibitor oligomycin (oligo) was added to determine leak respiration. Subsequent, addition of the mitochondrial membrane potential uncoupler FCCP resulted in maximal uncoupled respiration. **(F)** Subtraction of LEAK from ROUTINE respiratory rates resulted in ADP-linked respiratory rates. **(G)** To determine oxygen consumption in permeabilized cells, cells were permeabilized with digitonin in the presence of complex I-linked substrates glutamate and malate, just before initiation of each run, after which compounds were added and incubated for 90 min. **(H)** ADP was added to determine ADP-driven respiration, subtraction of non-mitochondrial respiration after addition of rot and AA was performed as described above. Statistical analysis: one-way ANOVA with Dunnett's *post hoc* analysis to compare values to control *p<0.05; **p<0.01; ***p<0.001. Mean ± SEM; n=3 biologically independent experiments.

Many previously reported AAC inhibitors have been associated with cellular and mitochondrial toxicity 43, 46, 48, 49, 55. A systematic comparison of their metabolic effects has not been performed. We assessed the effects of direct exposure of eighteen previously reported and commercially-available AAC inhibitors on cellular respiration in human HeLa cells (**Figure 1A**). All compounds were tested at a 100 μM, as concentrations of at least 200-fold plasma C_max_ demonstrated a more sensitive and specific prediction of the toxicity observed in humans 70. The canonical AAC inhibitors bongkrekic acid (BKA) and carboxyatractyloside (CATR) were included, and a strong and immediate inhibition of routine respiration could be observed with BKA, which is membrane permeable, but not with CATR (**Figure 1B**). Of the eighteen compounds tested, only CD-437, equisetin, ethacrynic acid, ibipinabant and tretinoin significantly inhibited respiratory capacity (**Figure 1C**). Addition of the mitochondrial uncoupler FCCP, to induce maximal respiration, exacerbated the inhibitory effect of most of these drugs, but did not reveal other compounds with a direct inhibitory effect (**Figure 1D**). Determination of the leak respiratory capacity (ADP-independent respiration; **Figure 1E**), enabled us to determine ADP-linked respiration, which was almost completely inhibited by BKA and CD-437 (**Figure 1F**). In addition, equisetin, ethacrynic acid, ibipinabant, and tretinoin significantly decreased ADP-linked oxygen consumption. The significant increased leak respiration by tretinoin (**Figure 1E**), which is also observed with indomethacin, is indicative of an increased proton leakage over the mitochondrial inner membrane. Therefore, the observed decrease in ADP-linked respiration could have been induced by an effect on mitochondrial coupling rather than directly on ADP-linked respiration.

To investigate whether any of the compounds may have been hindered by the plasma membrane, we permeabilized the cells (**Figure 1A**). Upon permeabilization, CATR completely inhibited ADP-driven respiration (**Figure 1G**). Moreover, permeabilization led to a significant respiratory inhibition by suramin and zidovudine (**Figure 1H**). Similar to the effects observed in intact cells (**Figure 1F**), equisetin and ethacrynic acid inhibited ADP-driven respiration (**Figure 1H**). Remarkably, ibipinabant and CD-437 did not inhibit ADP-driven respiration in permeabilized cells, whereas they did in intact cells, indicating an indirect effect.

The observed inhibition of respiration was shown to be a valuable measure of the cellular metabolic impact of the proposed AAC inhibitors. However, this remains a complex model system, illustrated by the mild inhibition of ADP-driven respiration in permeabilized cells. Therefore, further analysis is required to assess AAC interactions and inhibition directly.

### Functional expression and purification of human AAC1 in *S. cerevisiae*

**Figure 2:**
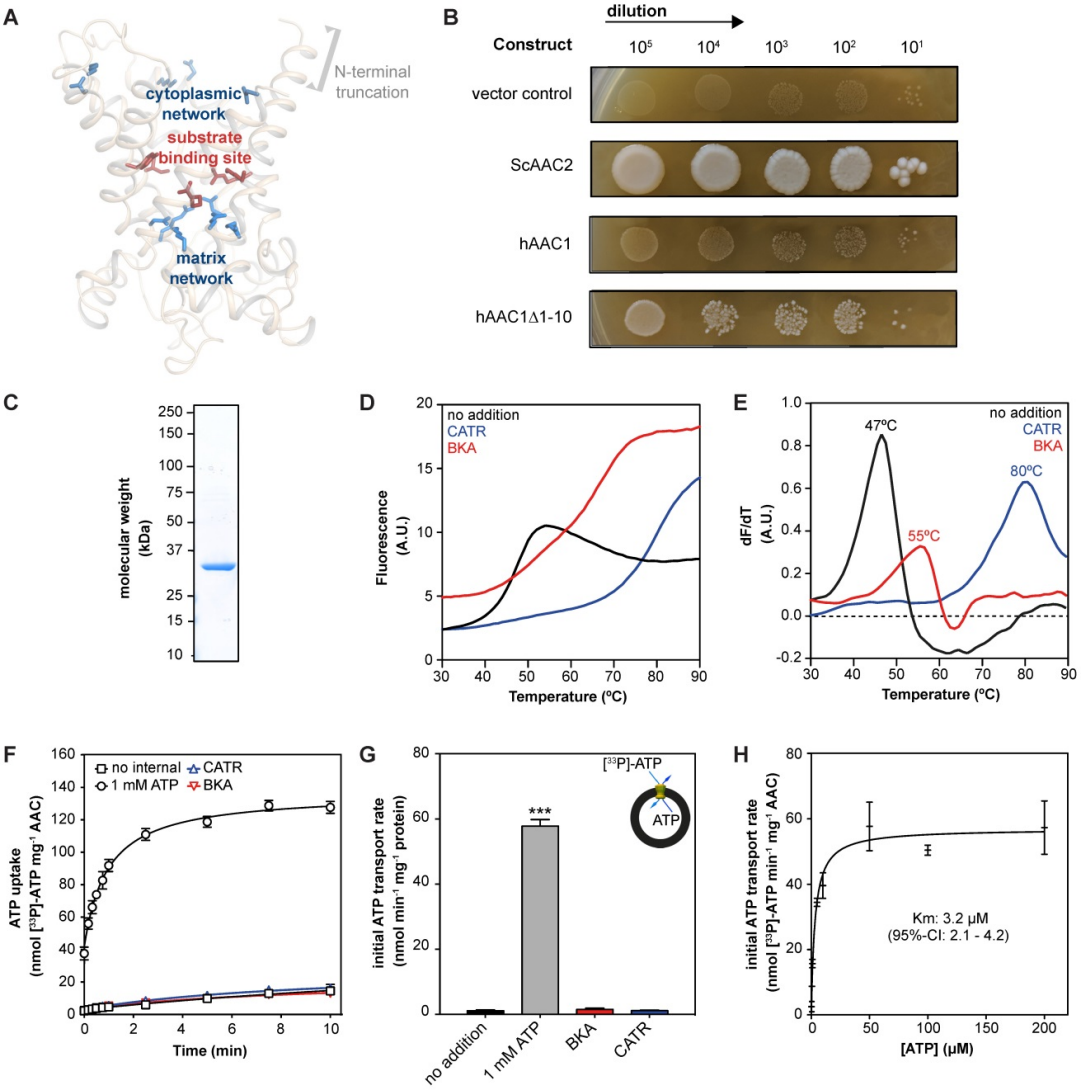
**| Expression and purification of human AAC1. (A)** Model of the N-terminal truncated protein hAAC1Δ1-10 showing that important functional elements are not affected by removal of the first 10 amino acids. **(B)** Functional complementation tests were carried out using a ten-fold serial dilution series, plated on YPG (a non-fermentable carbon source), and incubated for 6 days at 30 °C. **(C)** Instant-blue stained SDS-PAGE gel of purified hAAC1Δ1-10. (**D**) Typical unfolding curves of 3 μg hAAC1Δ1-10 in the absence and presence of 10 μM BKA (red line) and 10 μM CATR (blue line). (**E**) The peak in the derivative of the unfolding curve (dF/dT) is the apparent melting temperature (Tm) **(F)** [^33^P]-ATP uptake curves of hAAC1Δ1-10 reconstituted into proteoliposomes loaded with or without 1 mM ATP in the absence or presence of 10 μM CATR (blue curve) or 10 μM BKA (red curve) **(G)** Initial transport rates, calculated from panel (F) **(H)** Kinetics of ATP uptake. The internal ATP concentration was 1 mM. The apparent Km was determined using the Michaelis-Menten function. Statistical analysis: one-way ANOVA with Dunnett's *post hoc* analysis to compare values to control *p<0.05; **p<0.01; ***p<0.001. Mean ± SD; n=4 biologically independent experiments.

To provide a more unambiguous and accurate method to assess and compare drug-induced AAC inhibition, we adapted previously used methods to purify detergent-solubilized human AAC 29, 30, 63, 68. The isolation and expression of the properly folded monomeric four human AAC isoforms in bacteria and yeast cells is known to be problematic 59, 71, 72. We codon-optimized the sequence and truncated the first 10 residues of human AAC1 (hAAC1Δ1-10). The truncation does not interfere with substrate binding, or any other important functional elements of the carrier, but might lead to increased expression levels (**Figure 2A** and**Figure S2**) 29, 73.

Carrier function was assessed by its capacity to complement non-fermentable growth of a double AAC deletant strain (WB.12, *∆AAC1*, *∆AAC2*) 73. Complementation with full length human AAC1 (hAAC1) could not rescue growth of the deletant strain (**Figure 2B**). Cellular growth could be recovered with the hAACΔ1-10, although not to the same extent as wild-type *S. cerevisiae* AAC2 (ScAAC2), demonstrating that the truncation variant was active as an ADP/ATP carrier. Subsequently, we optimized the protein purification, resulting a yield of ~1.3 mg protein per gram of mitochondria, which provides sufficient protein to probe interactions with AAC in thermostability and transport assays (**Figure 2C**).

Next, we used thermostability analysis to evaluate protein stability. We monitored the unfolding of the protein population in a temperature ramp in the presence of the fluorescent dye *N*-[4-(7-diethylamino-4-methyl-3-coumarinyl)phenyl] maleimide (CPM), which binds to four cysteines buried inside the carrier that become solvent accessible upon protein unfolding. Consistent with previous thermostability analysis of ScAAC2 and TtAAC 34, 68, sigmoidal unfolding profiles were obtained for hAAC1Δ1-10 (**Figure 2D**). First-order derivatives showed a single peak, with an apparent melting temperature (Tm) of 47.0 ± 1 °C (**Figure 2E**), which represents the temperature at which approximately half of the population is unfolded. This value is similar to those observed for other AACs, such as ScAAC2 (44.7 °C) and TtAAC (49.5 °C) 68, which shows that the detergent-solubilized protein is folded and stable. Moreover, binding of canonical AAC inhibitors BKA and CATR lead to an increase in apparent melting temperature to 55 ± 1 °C and 80 ± 3 °C respectively (**Figure 2E**), as observed for other orthologs 68.

We next reconstituted human AAC1 into proteoliposomes, which allowed us to measure transport activity directly. CATR- and BKA-inhibitable [^33^P]-ATP/ATP transport activity, in which internal unlabeled ATP was exchanged for external radiolabeled ATP, was observed, but no activity was seen in the control assay (**Figure 2F-G**). Finally, we determined an apparent Km of 3.2 μM (95%-confidence interval 2.1 - 4.2 μM) (**Figure 2H**).

This apparent Km of transport is consistent with previously a published value using hAAC1 expressed and assayed in intact yeast mitochondria (Km^ADP^ 3.7 μM) 58. Moreover, it is in the same low micromolar range as the Km determined using hAAC fused to the periplastic maltose binding protein expressed in *E. coli* (Km^ATP^ 23.7 μM) 60*,* as the latter Km may be slightly higher due to interference of the periplastic maltose binding protein. Taken together, our work provides direct evidence that the reconstituted human AAC1 is stable, functional and displays the expected characteristics of the mitochondrial AAC.

### Drug-carrier interactions induce thermostability shifts of the human AAC1

Ligands are recognized by transport proteins through specific interactions. The formation of these additional bonds leads to an increase in the total number of interactions, resulting in an overall increase in the stability of the ligand-bound species compared to the unliganded species. This can be detected as a change in the apparent melting temperature of a protein population 74. We have shown that inhibitors of transport proteins cause an increase in protein thermostability 68, 74, 75. Furthermore, we have used thermostability shift analyses to identify substrates of a range of different transport proteins using this same principle 74. Compared to transport assays, thermostability analyses are high-throughput and relatively inexpensive.

**Figure 3:**
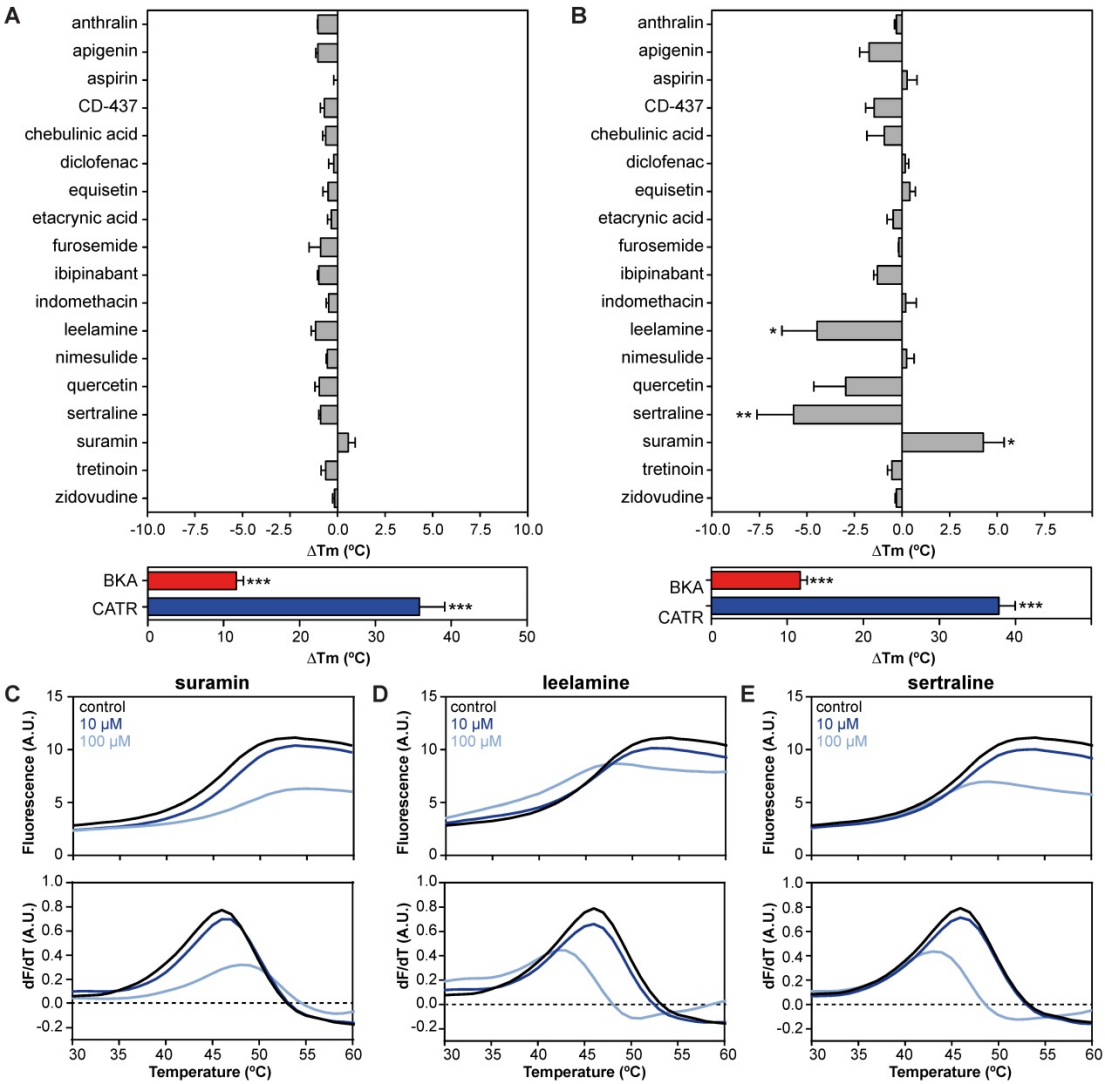
**| Drug-carrier interactions induce specific thermostability shifts of the human AAC1. (A)** The difference in melting temperature (ΔTm) is calculated by subtraction of the apparent melting temperature in the absence of compound from the one in its presence. The effect on stability of all compounds was determined at **(A)** 10 and **(B)** 100 μM in the presence of 5 μM ADP. Individual melting curves (upper panels) and first order derivatives (lower panels) of **(C)** suramin, **(D)** leelamine, **(E)** sertraline, which significantly altered the thermostability of the hAAC1Δ1-10. No addition (black trace), 10 μM compound (dark blue trace), 100 μM compound (light blue trace). Statistical analysis: one-way ANOVA with Dunnett's *post hoc* analysis to compare values to control *p<0.05; **p<0.01; ***p<0.001. Mean ± SEM; n=3 biologically independent experiments.

We evaluated 18 commonly used drugs and experimental compounds previously reported to inhibit AAC. As expected, the addition of both CATR (ΔTm; 35.8 ± 3.4 °C) and BKA (ΔTm; 11.7 ± 0.9 °C) resulted in a large increase in protein stability relative to no-inhibitor control assays (**Figure 3A**). Remarkably, none of the previously reported AAC inhibitors resulted in a statistically significant change in thermal stability at 10 μM (**Figure 3A**). However, at 100 μM, suramin stabilized the carrier significantly by 4.3 ± 1.1 °C (**Figure 3B** and** 3C**). In addition, thermal shift screening experiments usually produce destabilizing hits (negative ∆Tm) as well as stabilizing hits (positive ∆Tm). Here we observed that sertraline (ΔTm; 5.7 ± 1.9 °C) and leelamine (ΔTm; 4.5 ± 1.8 °C) destabilize AAC (**Figure 3D-E**). In the presence of 1 mM CD-437 a statistically significant destabilization was also observed (**Figure S1**). Although this method could benefit from using increasing concentrations of the compounds, it is not always possible due to quenching of the fluorescent signal.

Thermostability shift assays can be used as an effective high-throughput method to limit the number of potential off-target candidates from large compound libraries, providing important insights about transporter-drug interactions.

### Drug-induced inhibition of AAC-mediated ATP transport

**Figure 4:**
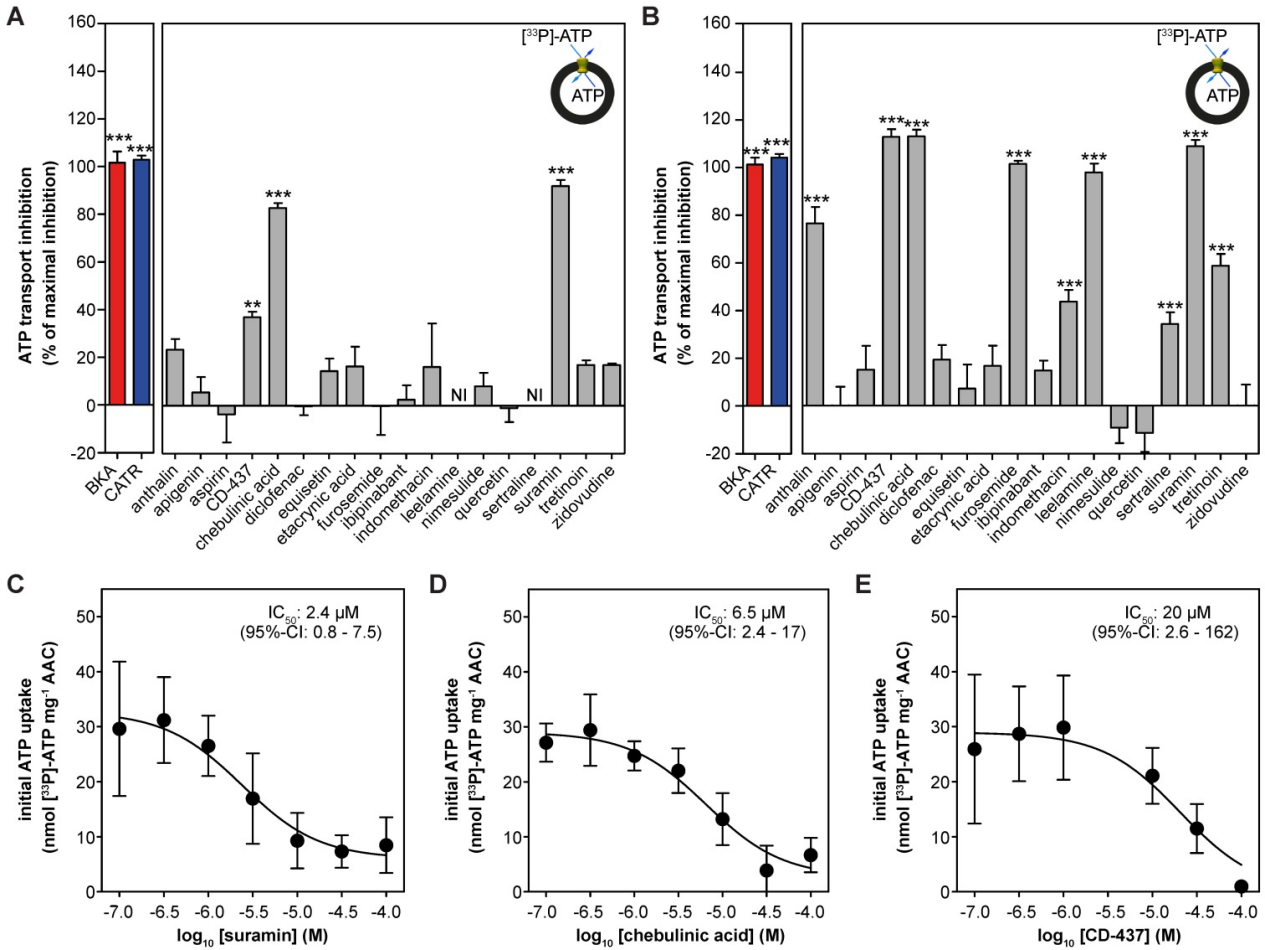
**| Drug-induced inhibition of AAC-mediated ATP transport.** The effect of proposed AAC inhibitors on [^33^P]-ATP uptake was monitored in proteoliposomes. Compounds were tested at **(A)** 10 or **(B)** 100 μM and were present internally and externally in the assay. Data are expressed as percentage inhibition, where 100% inhibition represents inhibition by CATR (blue). NI: not interpretable, due to extreme variability of the observed effects, as both inhibition and stimulation could be detected, even with six replicates. **(C-E)** IC_50_ values were calculated for **(C)** suramin, **(D)** chebulinic acid, and **(E)** CD-437. Statistical analysis: one-way ANOVA with Dunnett's *post hoc* analysis to compare values to control *p<0.05; **p<0.01; ***p<0.001. Mean ± SEM; n = 6 biologically independent experiments.

Next, we reconstituted human AAC1 into proteoliposomes to investigate the inhibitory effects of the proposed inhibitors directly. As expected, CATR and BKA fully inhibited AAC-mediated ATP transport at both 10 μM and 100 μM (**Figure 4A-B**). At 10 μM only suramin (92 ± 3%), chebulinic acid (83 ± 2%) and CD-437 (37 ± 2%) showed statistically-significant inhibition of ATP transport, relative to the inhibition observed with CATR (defined as 100%) (**Figure 4A**). Of note, leelamine and sertraline showed exceptional variability in transport rates (both stimulation and inhibition), which did not allow us to plot these rates with confidence. In addition to suramin, chebulinic acid and CD-437, furosemide and leelamine showed complete inhibition of ATP transport at 100 μM (**Figure 4B**). Statistically significant inhibition was also observed with anthralin (77 ± 7%), indomethacin (44 ± 5%), sertraline (34 ± 5%) and tretinoin (59 ± 5%). Finally, we determined IC_50_-values for the three most potent inhibitors. For suramin (2.4 μM; 95%-confidence interval, 0.8 - 7.5 μM) and chebulinic acid (6.5 μM; 95%-confidence interval, 2.4 - 17 μM) their inhibitory potential is comparable to previously observed IC_50_-values in hAAC2 (2.1 and 0.3 µM) 55. The inhibitory potential observed for CD-437 (20 μM; 95%-confidence interval, 2.6 - 162 μM) (**Figure 4C-E**) is also comparable to previously observed IC_50_-values for all human AAC isoforms expressed in yeast (9.5 - 22 µM) 49.

### Molecular surface charge and hydrophobicity are shared and important inhibitor characteristics

**Figure 5:**
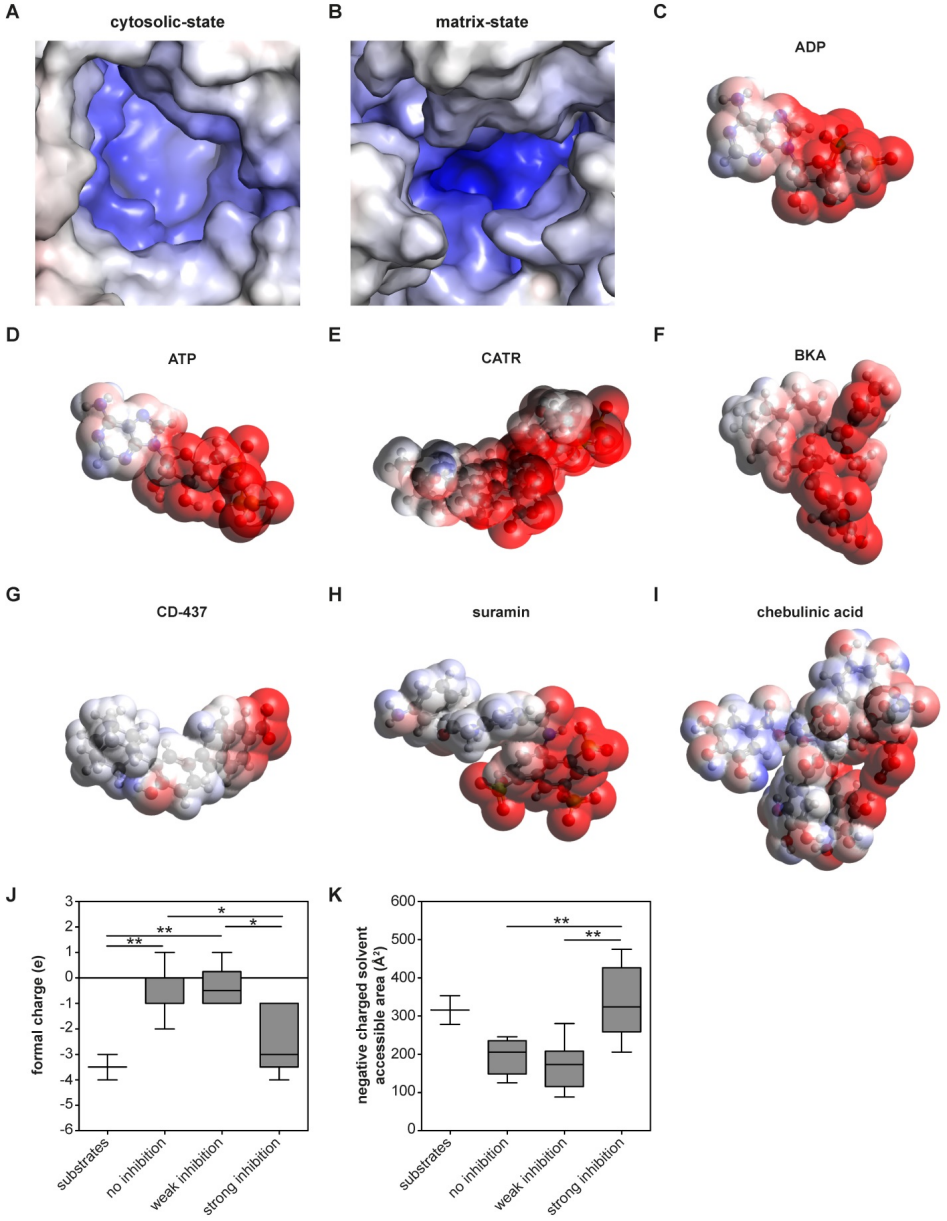
**| Molecular surface charge distribution are shared inhibitor and substrate characteristics. (A-B)** The electrostatic potential surface is shown for the AAC binding pocket. **(A)** The cytosolic-state model is based on the CATR-inhibited ScAAC2 structure (PBB: 4C9G) 63 and **(B)** the matrix-state on the BKA-inhibited TtAAC structure (PDB: 6GCI) 29. Surface is colored by electrostatic potential (blue, +20 kT e^-1^; white, neutral; red, -20 kT e^-1^). Surface charge distributions are shown for the AAC substrates **(C)** ADP and **(D)** ATP, the canonical AAC inhibitors **(E)** CATR and **(F)** BKA, as well as other AAC inhibitors **(G)** CD-437, **(H)** suramin and **(I)** chebulinic acid. Surface is colored by electrostatic potential (blue, +15 kT e^-1^; white, neutral; red, -15 kT e^-1^). Molecular surface calculations were performed at pH 7.0 for all substrates and inhibitors, and included the **(J)** formal charge and **(K)** negative charged solvent accessible area. Compounds were classified either as strong inhibitors (inhibition at 10 and 100 µM, **Figure 4A-B**), weak inhibitors (only inhibition at 100 µM, Figure 4B) or non-binders. Substrates are ADP and ATP. Statistical analysis: one-way ANOVA with Bonferroni's *post hoc* analysis to compare all conditions *p<0.05; **p<0.01. Data presented as Box-Whisker plots.

These analyses also allow us to identify which physicochemical properties of a compound are important for AAC inhibition. Previous observations have shown that almost all regions of the carrier are embedded in the inner membrane, which leaves nearly no exposed surfaces other than the central cavity 29, 63. Thus, this is the most likely site of binding for compounds. Moreover, the largely positively charged binding pocket is primed to bind the negatively-charged substrates ADP and ATP 29 (**Figure 5A-D**). Consistent with this, both canonical AAC inhibitors BKA and CATR are negatively charged, with formal charges of -3 and -4, respectively (**Figure 5E-F**). A similar charge distribution could be observed with the strong AAC inhibitors suramin and CD-437 (**Figure 5G-H**), which may suggest a similar mode of interaction. A more heterogeneous distribution of negative charges could be observed with the inhibitor chebulinic acid (**Figure 5I**). To quantify the relevance of this negatively charged area for AAC inhibitors further, we categorized them as non-binders, weak inhibitors (only inhibition at 100 µM, **Figure 4B**) or strong inhibitors (inhibition at 10 and 100 µM, **Figure 4A-B**). Compounds that did not or only weakly inhibit AAC have significantly less negative charge, whereas the formal charge of strong inhibitors is similar to that of the substrates ADP and ATP (**Figure 5J**). To investigate whether these negative charges are all solvent exposed, which is an important property for their interaction with AAC, we calculated the negatively charged solvent exposed surface area. This area was significantly larger in strong inhibitors compared to compounds that did not or only weakly inhibit AAC (**Figure 5K**). Collectively, this suggests that potent AAC inhibitors consist of a negatively charged region and a hydrophobic region, as observed in the substrates ADP and ATP.

## Discussion

A steadily increasing number of commonly prescribed drugs are known to interfere with mitochondrial function, but the underlying molecular mechanisms are largely unknown. Mitochondrial transport proteins form a significant class of potential mitochondrial off-targets, but are seldom considered. So far, most drug interactions with these transport proteins have been reported for mitochondrial AAC, but they have never been validated. Here, we describe the purification and functional reconstitution of hAAC1, which provides a direct tool to study drug-carrier interactions. We have used thermostability assays to investigate binding of hAAC1 for a subset of high affinity inhibitors, and inhibition kinetics using transport assays with hAAC1 reconstituted in proteoliposomes, which identified a wider range of inhibitors. Collectively, chebulinic acid, CD-437 and suramin were identified as the most potent inhibitors of AAC, with IC_50_-values in the low micromolar range. Therefore, these compounds showed a comparable inhibitory potency compared to BKA (2.0 µM) 55, but still far less potent compared to CATR for which IC_50_-values are in the low-nanomolar range 76. Combined with the inhibitory effects of suramin on respiratory capacity in intact cells, this agrees with the many adverse drug effects observed with suramin, including adrenal insufficiency, anemia and peripheral neuropathy 77. Another six compounds inhibited at 100 µM, but at this concentration, they are unlikely to be physiologically relevant. Finally, we showed that the presence of a negatively charged surface area associates with inhibitor strength. Remarkably, half of all previously published AAC inhibitors do not show significant inhibition in our assays, even at 100 µM, so they were false positives or exert their action on mitochondrial respiration via indirect effects.

The results obtained for suramin and chebulinic acid are consistent with previously reported data, obtained using hAAC2 expressed in inclusion bodies, refolded and reconstituted into proteoliposomes 55. CD-437 was shown to inhibit ADP uptake in isolated yeast mitochondria and *L. lactis* overexpressing all four human isoforms 49. CD-437 is a retinoid, which may point at a compound class inhibitory effect, as AAC inhibition was also observed with the prototypic retinoid tretinoin 44. Notably, leelamine demonstrated variable effects on transport, which is in agreement with the high variability at a 10 μM concentration in this study 49. It is important to note that many of the compounds that showed no inhibition in this study were previously proposed to inhibit AAC based on measurements of ADP/ATP exchange in intact bovine or rat mitochondria 39-45, 47, 48, 54. All these methods rely on functional oxidative phosphorylation, as well as other central metabolic pathways to drive ADP/ATP exchange. These drugs may inhibit these other systems, leading indirectly to decreased ADP and ATP transport rates. This notion is also supported by our assessment of the respiratory capacity, which demonstrates that both equisetin and ethacrynic acid are significant inhibitors of all respiratory states assessed, but they did not inhibit AAC-mediated ATP transport. These false positives illustrate the importance of using direct experimental systems. Importantly, respiratory capacity, especially ADP-linked and ADP-dependent oxygen consumption rates, was not affected by all AAC inhibitors, as chebulinic acid, furosemide and indomethacin did not inhibit either. For chebulinic acid this could potentially be explained by a poor mitochondrial uptake, due to high hydrophilicity. Overall, assessment of the cellular respiratory capacity does not seem to provide a valid measure of the capacity of a drug to inhibit AAC, but offers a method to evaluate the effects of these compounds on cellular metabolism as a whole.

In line with enhanced thermostability with the canonical AAC inhibitors BKA and CATR, which have also been observed previously 29, 34, 68, suramin increased AAC stability. In addition, the observed dose-dependent increase of stability with suramin correlated with transport inhibition (**Figure 3A-B**; **Figure 4A-B**). Even though hAAC inhibited with both leelamine and sertraline displayed decreased thermostability, they did inhibit AAC-mediated ATP transport, showing that both stabilizers and destabilizers need to be considered. None of the other compounds that inhibited transport at high concentrations, such as anthralin, chebulinic acid, furosemide, indomethacin, and tretinoin, caused a significant shift in thermostability. Although there might some technical issues, *e.g.* interference with the fluorescent signal with chebulinic acid, these data may indicate that they are not strong binders. Similarly, the difference in thermal shifts observed between suramin and BKA, which have comparable IC_50_-values (2.4 and 2.0 µM) in transport assays, shows that these assays probe fundamentally different properties of the protein in relation to the binding of these compounds. The former tests the number and strength of interactions at high temperatures, when the carrier is resuspended in detergent solution, whereas the latter tests the effect of the compound on the transport cycle at room temperature, when the carrier is incorporated in the membrane. At 1 mM we observed a destabilization effect with CD-437 (**Figure S1**), which supports the idea that higher concentrations are needed to see an effect on protein thermostability relative to the concentrations needed to observe inhibition in transport assays. Transport assays using reconstituted AAC provide the most straightforward and sensitive experimental approach to evaluate and compare drug-induced inhibition, even though they are low-throughput and expensive compared to thermostability assays, which provide a more practical and fast approach to identify strongly binding AAC inhibitors from libraries of compounds. It is important to note that the most potent inhibitor of AAC-mediated ATP transport, suramin, also induced stabilization of hAAC in our thermostability shift assays.

Previously, comparison of the different human AAC isoforms demonstrated only minor differences in IC_50_-values, including for CD-437 and leelamine 49. This observation can be explained by the conserved sequence homology and structural similarities of these different isoforms, which all share the three contact points of the substrate binding site 78, and many other functionally important features, such as the matrix salt bridge network 33, 63, cytoplasmic salt bridge network 29, 34, 63, 78, the glutamine braces 63, tyrosine braces 29, and motifs involving small amino acid residues 1, 29, 31 (**Figure S2**). Consequently, compounds that inhibited hAAC1 in our study can also be expected to inhibit the other human AAC isoforms. This idea is emphasized by the comparable inhibitory potency (IC_50_-values) we observed for chebulinic acid and suramin in hAAC1 (6.0 and 2.4 µM) with those previously observed in hAAC2 (2.1 and 0.3 µM) 55.

In conclusion, we have provided initial characterization of the human AAC1 and demonstrated its relevance to study drug-induced inhibition. Moreover, the ability to compare compounds within the same experimental system, including transport assays using reconstituted, detergent-purified protein, enabled us to gain new insights into the chemical compound features important for inhibition. Many other mitochondrial transport proteins are off-targets for commonly prescribed drugs, such as the mitochondrial pyruvate carrier, which is inhibited by thiazolidinediones 79, 80 and the mitochondrial dicarboxylate carrier, which is inhibited by cephalosporin antibiotics 81. Therefore, transport proteins can play a pivotal role in drug-induced mitochondrial dysfunction. Consequently, similar experimental approaches are also warranted for these and other mitochondrial transport proteins. Eventually, these analyses will provide insights in the role of mitochondrial transport proteins in drug-induced mitochondrial dysfunction and will thereby contribute to the development of drugs with an enhanced safety profile.

## Supplementary Material

Supplementary figures.

## Abbreviations

AA: antimycin A
AAC: ADP/ATP carrier
ABC: ATP-binding cassette
ADP: adenosine diphosphate
ATP: adenosine triphosphate
BKA: bongkrekic acid
CATR: carboxyatractyloside
CI: confidence interval
CPM: *N*-[4-(7-diethylamino-4-methyl-3-coumarinyl)phenyl] maleimide
CV: column volume
DMEM: Dulbecco's modified Eagle's medium
DMSO: dimethylsulfoxide
FCCP: carbonyl cyanide-*4*-(trifluoromethoxy)phenylhydrazone
FCS: fetal calf serum
MAS: mitochondrial assay solution
NI: not interpretable
OCR: oxygen consumption rate
oligo: oligomycin
PCR: poly chain reaction
PDB: protein databank
PEG: polyethylene glycol
rot: rotenone
SEM: standard error of the mean
SLC: solute carrier
YEPG: yeast extract peptone glycerol
YPG: yeast peptone glycerol
