# Supplementary material for: Characterization of drug-induced human mitochondrial ADP/ATP carrier inhibition
Source: Theranostics. 2021 Mar 5;11(11):5077–91. doi: 10.7150/thno.54936 (PMC8039944; doi:10.7150/thno.54936)
Supplement: Supplementary file 1 — Supplementary figures. [file thnov11p5077s1.pdf]

SUPPLEMENTARY MATERIALS

Characterization of drug-induced human mitochondrial ADP/ATP carrier inhibition

Stephany Jaiquel Baron, Martin S. King, Edmund R.S. Kunji, Tom J.J. Schirris

Content:

Supplementary Figures 1 - 2

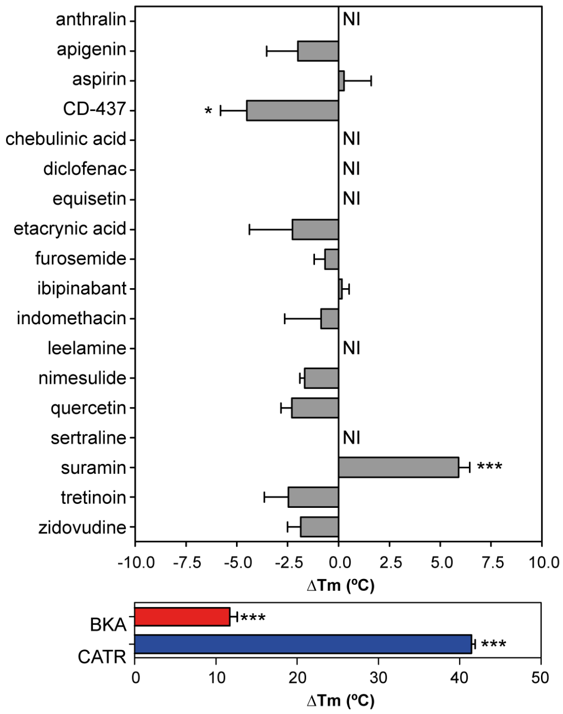

Figure S1 | As figure 3 legend, except compounds are at 1 mM. NI: not interpretable, due to interference of the compounds with the fluorescent measurement. Statistical analysis: one-way ANOVA with Dunnett's *post hoc* analysis to compare values to control *$p<0.05$; ***$p<0.001$. Mean ± SEM; n=3 biologically independent experiments.

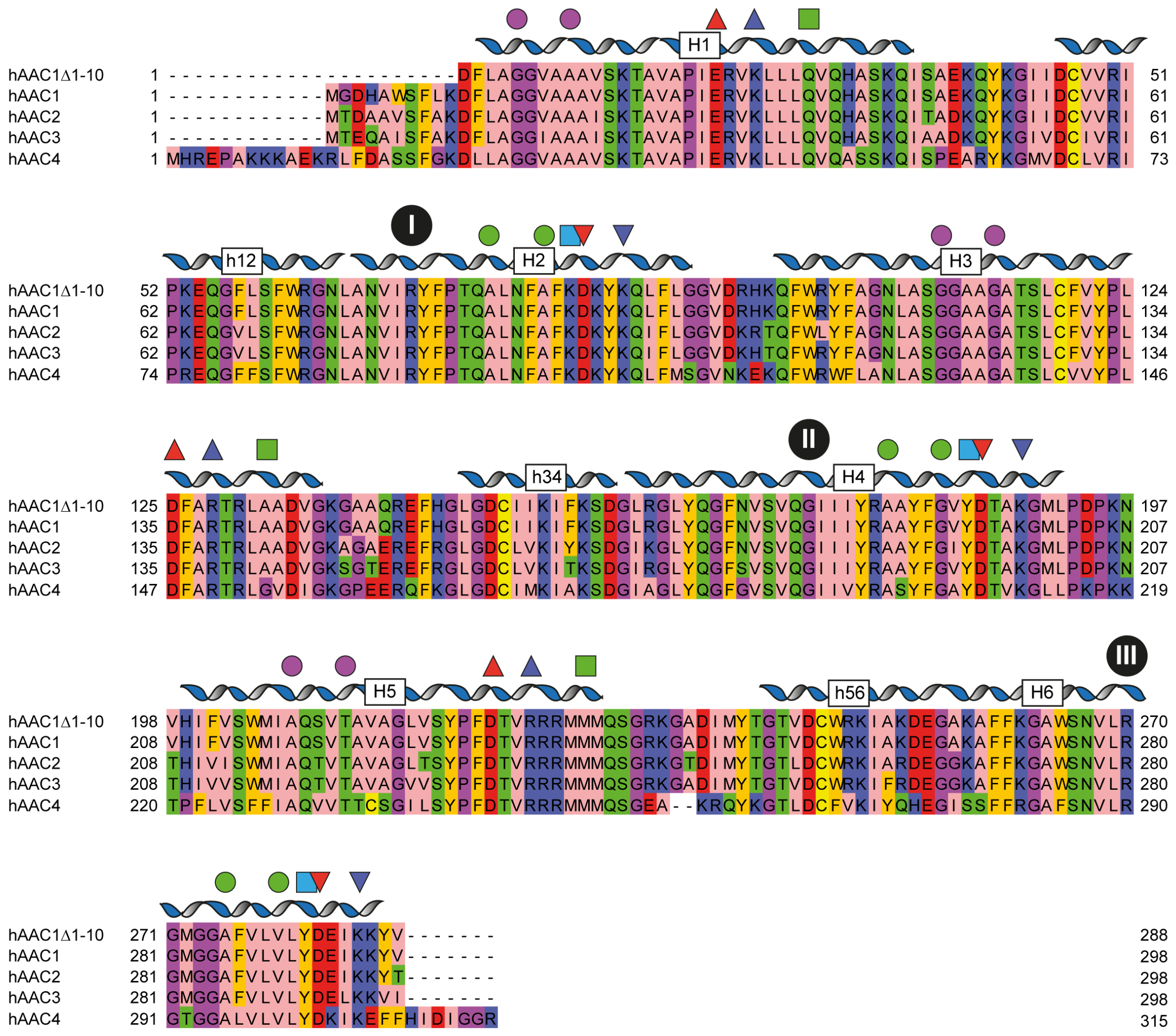

Figure S2 | Alignment of amino acid sequences of all human AAC isoforms. Alignment of the truncated hAAC1Δ1-10 and all four human AAC isoforms (UniProt entry codes: hAAC1, P12235; hAAC2, P05141; hAAC3, P12236; hAAC4, Q9H0C2). Amino acids are colored according to their properties: basic K, R and H are blue, acidic D and E are red, polar N, Q, S and T are green, aliphatic A, I, L,M and V are pink, aromatic F, Y and W are orange, structural G and P are magenta, and C is yellow. The negatively charged (red) and positively charged (blue) residues of the matrix and cytoplasmic networks are indicated by up and down triangles, respectively. The positions of the glutamine and tyrosine brace are indicated by green and cyan squares, respectively, even if they are not conserved in AACs. The purple and lime circles indicate the positions of the GxxxG and $\pi xxx\pi$ motifs. The contact points of the substrate binding site are shown in black circles with roman numerals [63].
